# Implementing Geographic Information System (GIS) tools: Algorithm and Quantum Geographic Information System (QGIS) plugin for automatic detection of riverbed forms

**DOI:** 10.1016/j.mex.2024.103041

**Published:** 2024-11-09

**Authors:** Klaudia Kryniecka

**Affiliations:** Faculty of Building Services, Hydro and Environmental Engineering, Warsaw University of Technology Nowowiejska 20St, Warsaw 00-653, Poland

**Keywords:** QGIS plugin, Sandbar detection, Riverbed forms, Sentinel 2, Water mask, Water indices, Sandbar Detector plugin for QGIS

## Abstract

This paper introduces the Sandbar Detector plugin for Quantum Geographic Information System (QGIS), designed to streamline the detection and analysis of riverbed forms, previously hindered by time-consuming manual methods requiring extensive expertise in remote sensing and Geographic Information System (GIS). The Sandbar Detector plugin, developed in Python, leverages the Sentinel Water Mask (SWM), a reliable remote sensing water index, for precise differentiation between water and land. By integrating SWM with QGIS, the plugin utilises high-resolution data from Sentinel-2 satellites, offering a robust tool for environmental analysis.

• Automation of detection: The plugin automates detecting riverbed forms, enhancing data analysis efficiency and consistency.
• User-friendly interface: It makes the plugin accessible to users without advanced remote sensing and GIS knowledge.
• High-resolution data: The plugin uses Sentinel-2 satellite data, ensuring precise and reliable results.

The plugin was tested on the Lower Vistula River, a central river system in Poland known for its dynamic riverbed forms shaped by natural and anthropogenic factors. The automation provided by the plugin reduces human error and supports more accurate environmental monitoring, which is crucial for better water resource management and conservation efforts. The Sandbar Detector plugin is freely available on GitHub, making it easy to access and use for collaborative research (Kryniecka, 2024).

## Graphical abstract

**Figure:**
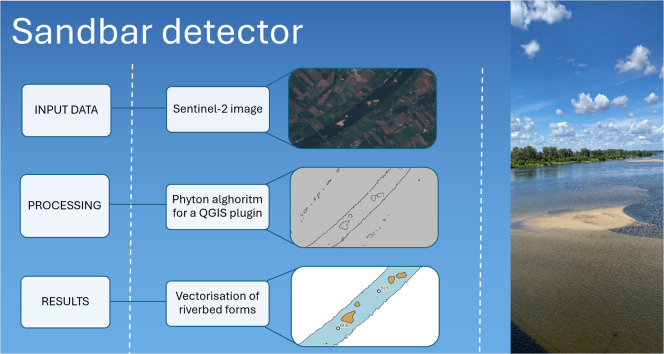

Specifications table

**Table:** 

Subject area:	Environmental Sciences
More specific subject area:	Developing and implementing a Python algorithm for automating the detection and analysis of riverbed forms within the QGIS environment.
Name of your method:	Sandbar Detector plugin for QGIS
Name and reference of original method:	Not applicable
Resource availability:	QGIS: https://qgis.org/en/site/forusers/download.html Sentinel images: https://www.sentinel-hub.com Sandbar detector plugin: https://github.com/KK-hydro/Sandbar-detector/

## Background

Existing methods for detecting riverbed forms, including sandbars [7,8] are performed manually using separate tools, a process is both time-consuming and required extensive expertise in remote sensing and Geographic Information System (GIS). Although effective, the manual method posed significant challenges due to its complexity and the extensive time required to process and analyse the data. Moreover, due to their inherent subjectivity, carry a higher risk of errors, whereas the automated approach introduces standardization, ensuring consistency and greater accuracy in the result. There are various automated tools available for image classification, these are primarily designed for land cover classification, such as distinguishing different types of vegetation. However, these tools are not specifically tailored for directly distinguishing between water and land in the context of riverbed form detection.

To address the challenge of automating the detection process of riverbed forms, the Sandbar Detector plugin for Quantum Geographic Information System (QGIS) was developed. Developed in Python, this plugin leverages the Sentinel Water Mask (SWM) for high-precision analysis. The SWM is a well-established remote sensing water index that provides reliable differentiation between water and land, developed by the Crisis Information Center team [12]. By integrating this advanced index into the QGIS environment, the plugin offers a robust tool for environmental analysis. This integration allows users to benefit from the high-resolution data from Sentinel-2 satellites, which is crucial for accurately detecting and classifying riverbed forms.

The analysis of riverbed forms is widely covered in international literature as these studies help clarify river dynamics and their response to environmental changes and human interventions [2,3,9], and the plugin facilitates a more comprehensive and extensive examination of these riverbed forms. Understanding these processes is crucial for water resource management and environmental conservation. Despite significant advances in geomorphology and hydrology, the dynamic nature of rivers and the limitations of traditional research methods often make precise observations challenging.

The Lower Vistula, as one of the most important and largest river systems in Poland, serves as an exceptional research area frequently mentioned in the literature [1,5,10]. The riverbed forms of the Lower Vistula, shaped over centuries by both natural processes and human activities, are dynamic indicators of the interactions between the river and its surrounding environment. Analysing the dynamics of these riverbed forms provides valuable insights into how the river adapts to changing hydrological conditions and helps understand human pressure's impacts on the river ecosystem.

Recognizing the challenges in manually detecting and analyzing riverbed forms the Sandbar Detector was developed to automate this previously time-consuming and specialized process.. This automation reduces the potential for human error and ensures that the results are consistent and reliable. The plugin's user-friendly interface further simplifies the process, making it accessible to users who may not have extensive experience in remote sensing or GIS.

## Method details

The developed method of automated riverbed forms detection offers a novel solution, significantly improving efficiency and accuracy compared to traditional manual techniques. Its main task is efficiently distinguishing land from water, utilising the SWM, remote sensing index for this purpose. The Sandbar Detector plugin is available on GitHub as open-source software (https://github.com/KK-hydro/Sandbar-detector/). This setup allows researchers to easily access the tool, customize it to fit their needs, and apply it to a range of research applications [6]. Its use in the algorithm translates into high accuracy in distinguishing water bodies, identifying them, and analysing riverbed forms. Thanks to its features, the Sandbar Detector plugin transforms satellite images into vectors that are easy to analyse and visualise within the GIS environment. The Sandbar Detector tool uses Sentinel-2 Level 1C satellite images, precisely four spectral bands (Blue, Green, NIR, and SWIR1), to which it is adapted. During processing, it calculates the SWM spectral water index, creates a binary water mask image, and results in a final vector layer product.

Python 3.9 was used to develop the plugin. Specific functions were developed to help collect and generate the necessary information for analysis to achieve the set goals. Python, as a programming language, is flexible and versatile, making it an ideal tool for creating GIS plugins. Its libraries, such as PyQt5, Pandas, NumPy, and GDAL, enable easy manipulation of spatial data and the creation of user interfaces. These tools allow for the creation of advanced geospatial analyses and automating data processing workflows. In the project, Python's capabilities were utilised to build functions that collect data from various sources, process it, and present it in a user-friendly format [11].

### Developed functions

• *raster2Calculator*: The *raster2Calculator* function returns collected input objects as *QgsRasterCalculatorEntry* data. The result of this function can then be easily used in *QgsRasterCalculator. QgsRasterCalculatorEntry* is a class that stores information about a single input raster, including its name, file path, and band index. This function is helpful when mathematical operations must be performed on various raster layers, using their bands as variables in the equation.
• *mapAlgebra*: The *mapAlgebra* function performs map algebra, utilising *QgsRasterCalculator* to
• conduct calculations on raster layers. Map algebra is a spatial analysis technique that allows manipulation of pixel values in one or several rasters to create a new raster. This can be achieved by applying various mathematical operations, such as addition, subtraction, multiplication, or division. *QgsRasterCalculator* enables the execution of complex mathematical expressions on raster layers, which is extremely useful in geographic and environmental analyses.
• *vectorise*: The vectorise function returns a resulting vector layer. It uses *gdal. Polygonise* to create vector
• polygons for all connected regions of pixels in the raster that share the same pixel value. *gdal. Polygonise* is a tool from the GDAL (Geospatial Data Abstraction Library), which is a library for translating raster and vector geospatial data formats. The vectorisation process is particularly useful when converting raster data (e.g., land classification maps) into vector data (polygons), allowing easier manipulation and spatial analysis of this data in *GIS.raster2Calculator*.

### General script

The initial step involves importing all the necessary modules and setting up global variables. For better modularity and organisation, to create the functions were opted in a separate file and imported them into the main script as a module.

First, the user provides the input layers. These layers are then processed using the raster2Calculator function. This function prepares the layers by converting the m into QgsRasterCalculatorEntries, making them suitable for further geospatial calculations.

Next, the *mapAlgebra* function performs map algebra operations on the prepared raster layers. By leveraging the *QgsRasterCalculator*, this function executes various geospatial calculations, such as reclassification, combination, and mathematical operations on the raster data, according to the analysis requirements. In the analysis, the Sentinel Water Mask index was calculated.

$$SWM=\frac{B2+B3}{B8+B11}$$

Where: B2 is Band 2 - BLUE, with a wavelength of 490 nm, B3 is Band 3 - GREEN with a wavelength of 560 nm, B8 is Band 8 - NIR with a wavelength of 842 nm and B11 is Band 11 - SWIR1 with a wavelength of 1610 nm.

Additionally, a binary map was created by subtracting a threshold value from the SWM, effectively distinguishing areas based on this threshold. The procedure involves dividing the pixels into two classes, if pixels with the same or similar value correspond to identical objects [4]. The first class consists of pixels with values ranging from 0 to 0.957, representing water in the image, while pixels above 0.957 correspond to land objects. The threshold value of 0.957 was taken from Kryniecka's work in 2021, where, using the pixel distribution histogram and based on the Otsu method [13], threshold values for various remote sensing indices, including the SWM index used in the plugin, were determined.

After the map algebra operations, the resulting raster data is converted into vector format using the vectorise function. This function employs gdal. Polygonise to generate vector polygons for regions of connected pixels that share the same value. This vectorisation process is crucial for transforming raster data into a format that can be more easily analysed and interpreted in subsequent steps.

Finally, the vectorised output is saved into a file specified by the user. This ensures that the processed geospatial data is stored conveniently for future use, analysis, or visualisation. Following these steps, the script effectively processes the geospatial data through well-defined and organised stages, ensuring a transparent and efficient workflow.

**Fig. 1:**
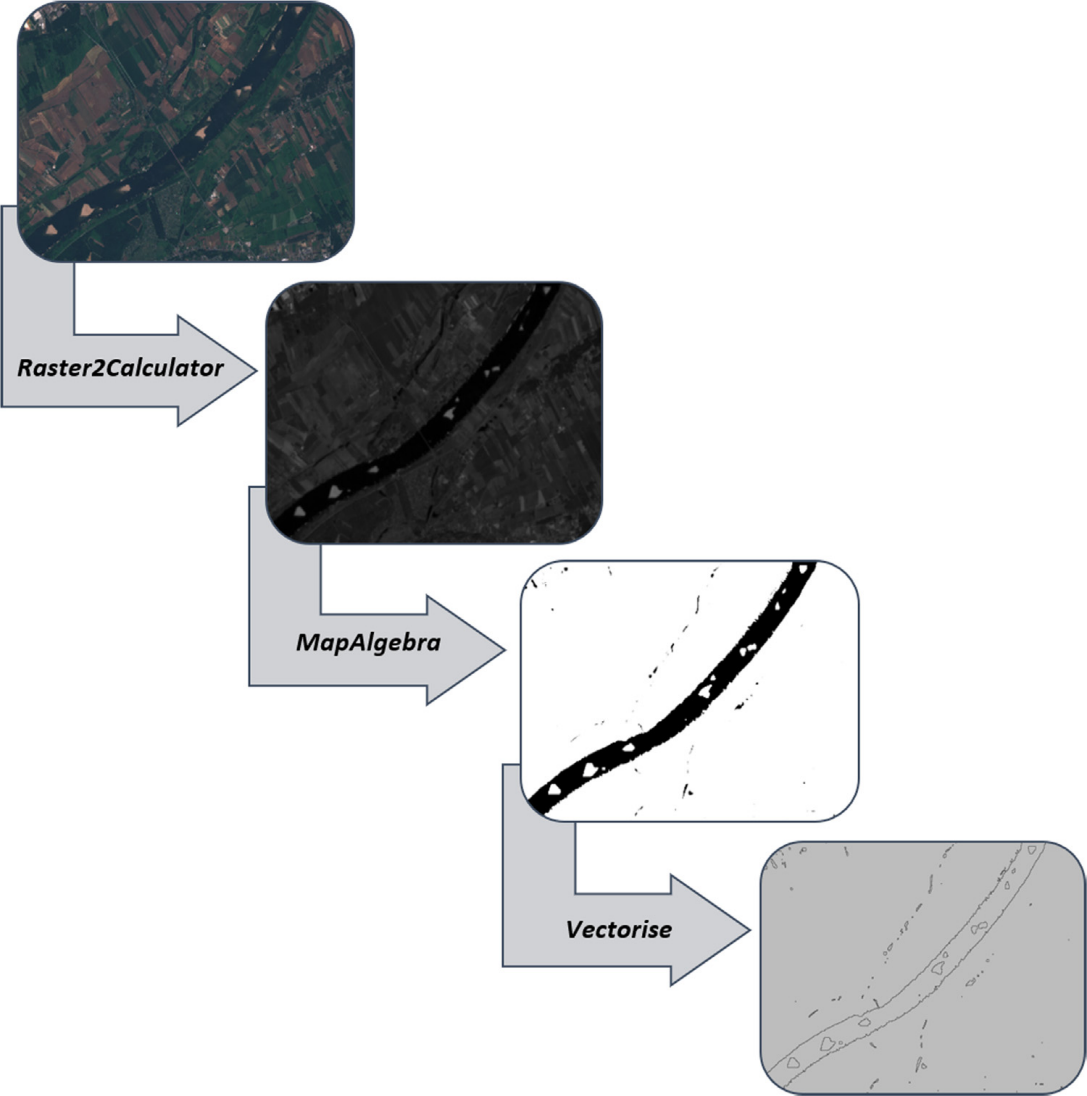
Workflow for Sentinel-2 satellite image processing using sandbar detector.

**Fig. 2:**
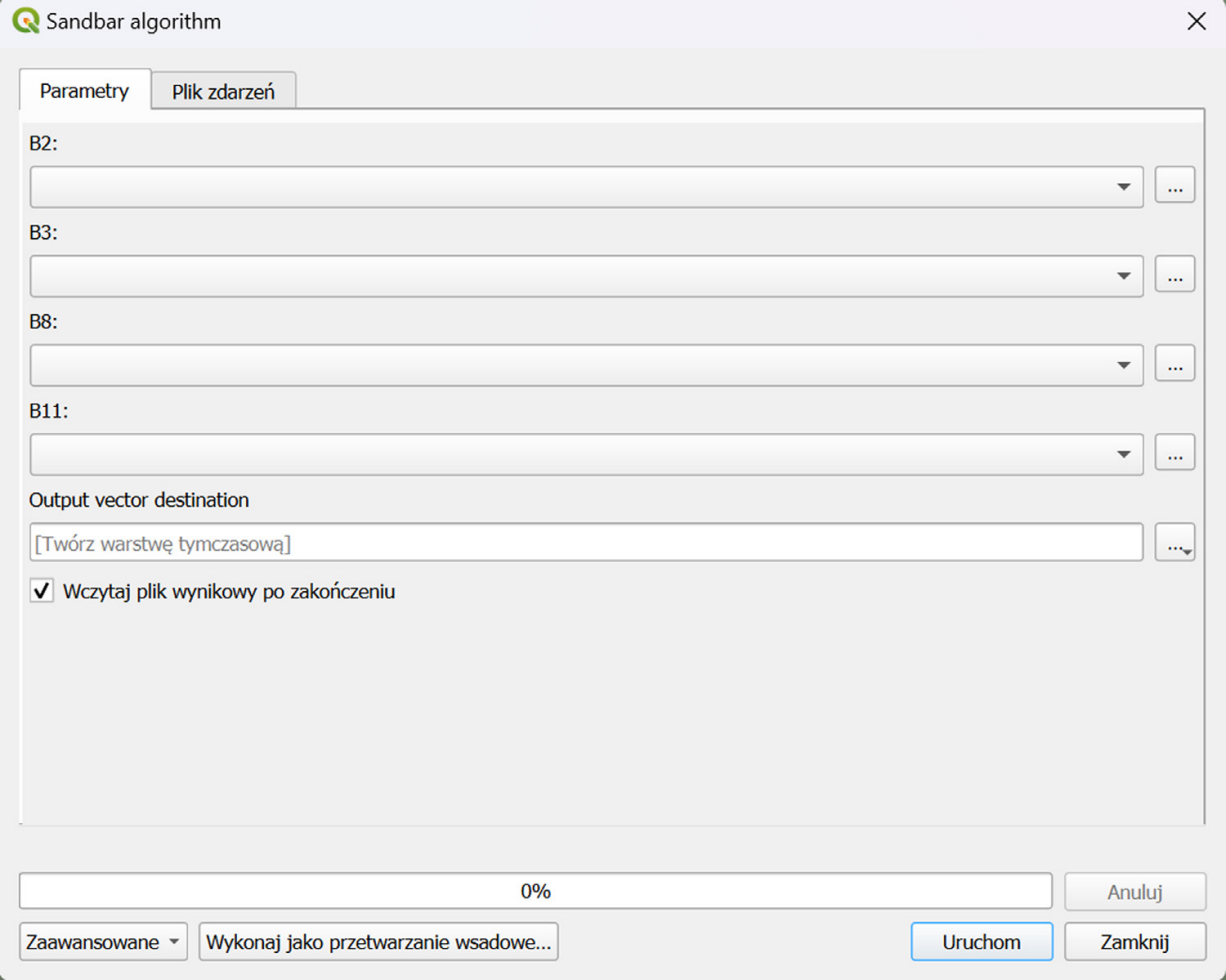
Sandbars Detector tool dialog window.

To use the Sandbars Detector tool, as shown in the dialogue window in Fig. 1, you need to import Bands 2, 3, 8, and 11 accordingly. Fig. 2

## Method validation

The development of the QGIS plugin was driven by necessity. In previous publications, detecting riverbed forms was described and analysed. However, to streamline this work, benefit other researchers, and promote remote sensing in hydrology, the decision was made to automate detecting sandbars.

The manual method using single tools in QGIS, while effective, was time-consuming and labour-intensive, requiring significant effort for data processing and analysis. Automating this process reduces the workload and increases the consistency and accuracy of the results. The QGIS plugin leverages remote sensing technology and advanced algorithms to quickly and efficiently identify and classify various riverbed forms.

By automating the detection process, the plugin enhances the capability to analyse large datasets and perform hydrology studies more efficiently. This is particularly beneficial for hydrologists and environmental scientists who need to monitor changes in river morphology over time. The plugin's ability to provide rapid and reliable data can lead to better-informed decision-making and more effective management of water resources.

**Fig. 3:**
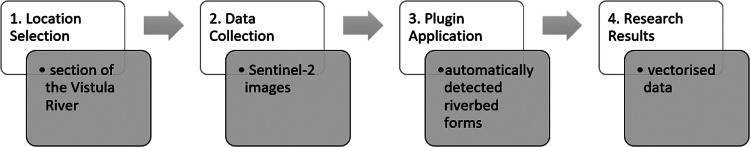
Procedure scheme.

The process of detecting riverbed forms is based on the method of water indices, which is widely used in the literature. According to studies by Kryniecka and Magnuszewski [7] conducted on the Lower Vistula River regarding detecting riverbed forms, using the SWM index provides the most effective and precise results for distinguishing water from land. A series of studies were conducted on selected river sections to validate the effectiveness of the automated method and the developed QGIS plugin. Fig. 3 illustrates the sequential process of converting satellite images into the final product using the specified algorithm, with detailed descriptions provided subsequently.

The study included the following steps:

1. Selection of location: River sections with diverse hydrological conditions were selected to test the universality of the method. This section of the Vistula River was chosen for its unique characteristics: dynamic hydrological conditions and various riverbed forms such as sandbars, islands, and ripples. This makes it an ideal site for testing the method's applicability.
2. Data collection: Sentinel-2 remote sensing data from the Sat4Envi platform was collected for the Lower Vistula section between kilometres 806 and 809 near the Chełmno water gauge. This high-resolution data was chosen from periods of minimal cloud cover and low water stage to ensure clarity and that sandbars were exposed. The selected data provided a clear and detailed view of the riverbed, essential for accurate detection and analysis.
3. Application of the plugin: The developed QGIS plugin automatically detected riverbed forms on the Sentinel-2 data. This plugin automated the detection of riverbed forms, processing the Sentinel-2 data to identify and classify features like sandbars. This automation increased efficiency and consistency compared to manual methods.
4. Research results: The results obtained using the QGIS plugin were compared with those obtained using traditional manual and SWM methods. Traditional manual methods involve drawing lines along the edges of islands and other features. This process is highly labour-intensive and requires significant expertise, making it time-consuming and prone to human error. The SWM method using QGIS tools involves a series of steps using various tools in QGIS based on the SWM. While more automated than manual methods, considerable manual intervention and specialised knowledge are still required to process the data accurately. A detailed comparison of the results is presented below, focusing on several key metrics, including accuracy, efficiency, and consistency.

**Fig. 4:**
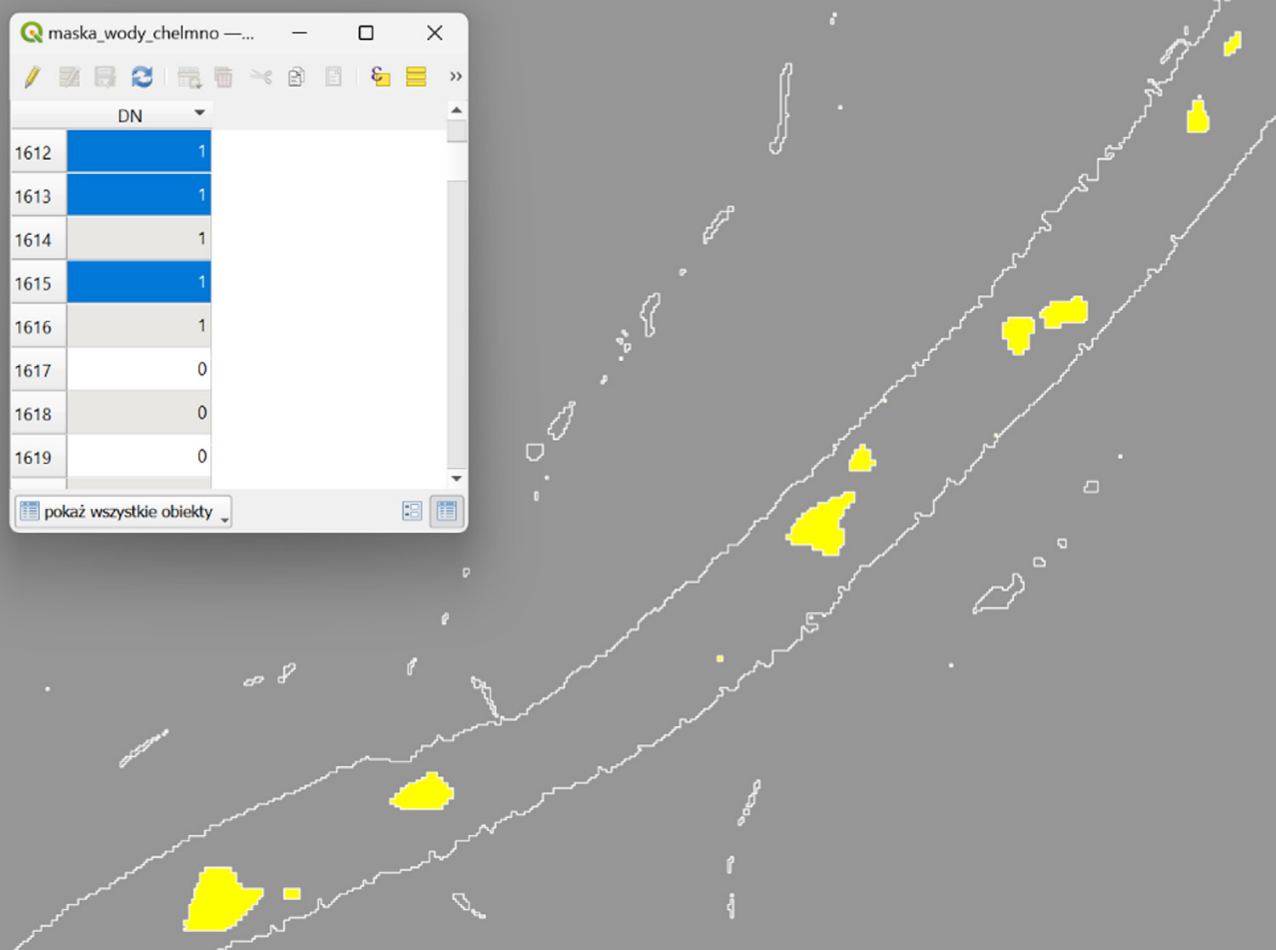
The Sandbar Detector plugin in QGIS generated the final vector product, along with a portion of the attribute table showing the differentiation between land and water vectors.

The image shown in Fig. 3 displays the final vector product generated by the Sandbar Detector plugin in QGIS. The vectors clearly distinguish between land and water features. This makes visual interpretation and further analysis within the QGIS environment easier. Accompanying the vector map is a portion of the attribute table detailing the classification of each vector element, including attributes such as type (land or water), area, and perimeter. Users can select and choose the elements of interest for further analysis. This comprehensive view showcases the plugin's capability to automatically and accurately identify riverbed forms, streamlining the environmental monitoring and analysis workflow. Fig. 4

**Fig. 5:**
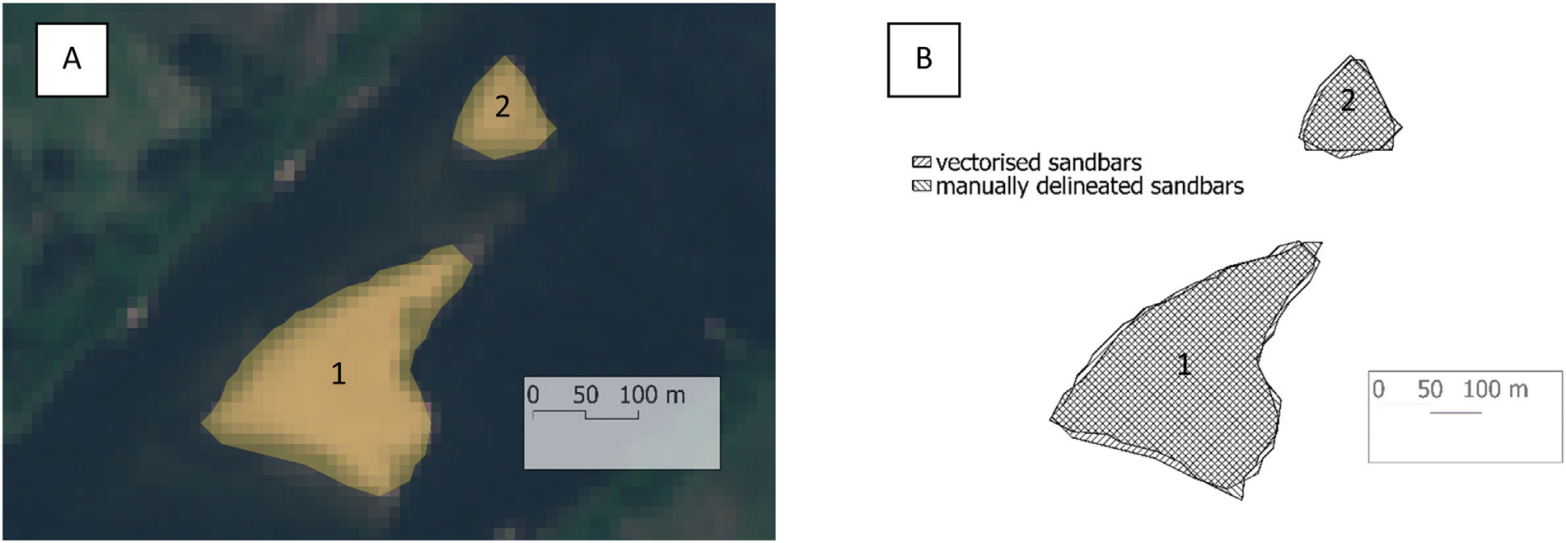
A) Comparison of true-colour satellite imagery with overlaid vector data representing sandbars, illustrating the relative size of the generated vector in comparison to real-world features. B) Comparison of two overlaid layers, one automatically generated and one manually delineated, to evaluate their respective sizes.

The QGIS plugin demonstrated high accuracy in detecting riverbed forms. The results showed a firm agreement with those obtained from traditional manual methods. The plugin's automated detection capabilities proved reliable, correctly identifying riverbed features such as sandbars and islands. This accuracy was validated by cross-referencing with true-color satellite imagery (Fig. 5. A). The QGIS plugin provided consistent results. This high repeatability is crucial for scientific research, ensuring the results are reliable and can be replicated in different studies. The consistency of the plugin's results was compared to the variability often seen in manual methods, which can be subject to human error and inconsistencies. In the RGB satellite image, the submerged part under the water is also visible, making it difficult for a human to distinguish the boundary between land and water. However, using this method, a precise pixel value is defined that delineates this boundary. To verify the results, manual tracing was performed on two riverbed forms, and the areas obtained from the manual tracing were compared to the vector outlines of the riverbed forms generated using the Sandbar Detector plugin (Fig. 5. B). After performing both manual and automated methods, the areas of the sandbars were calculated (table 1). The results are closely matched, demonstrating the reliability and precision of the automated method.

**Table 1:** Comparison of sandbar areas obtained using two different methods.

No. sandbar	Manually method [m^2^]	Automatic method [m^2^]	Difference [m^2^]
1	30,792.04	32,634.26	1842.22
2	5399.31	5872.16	472.85

**Fig. 6:**
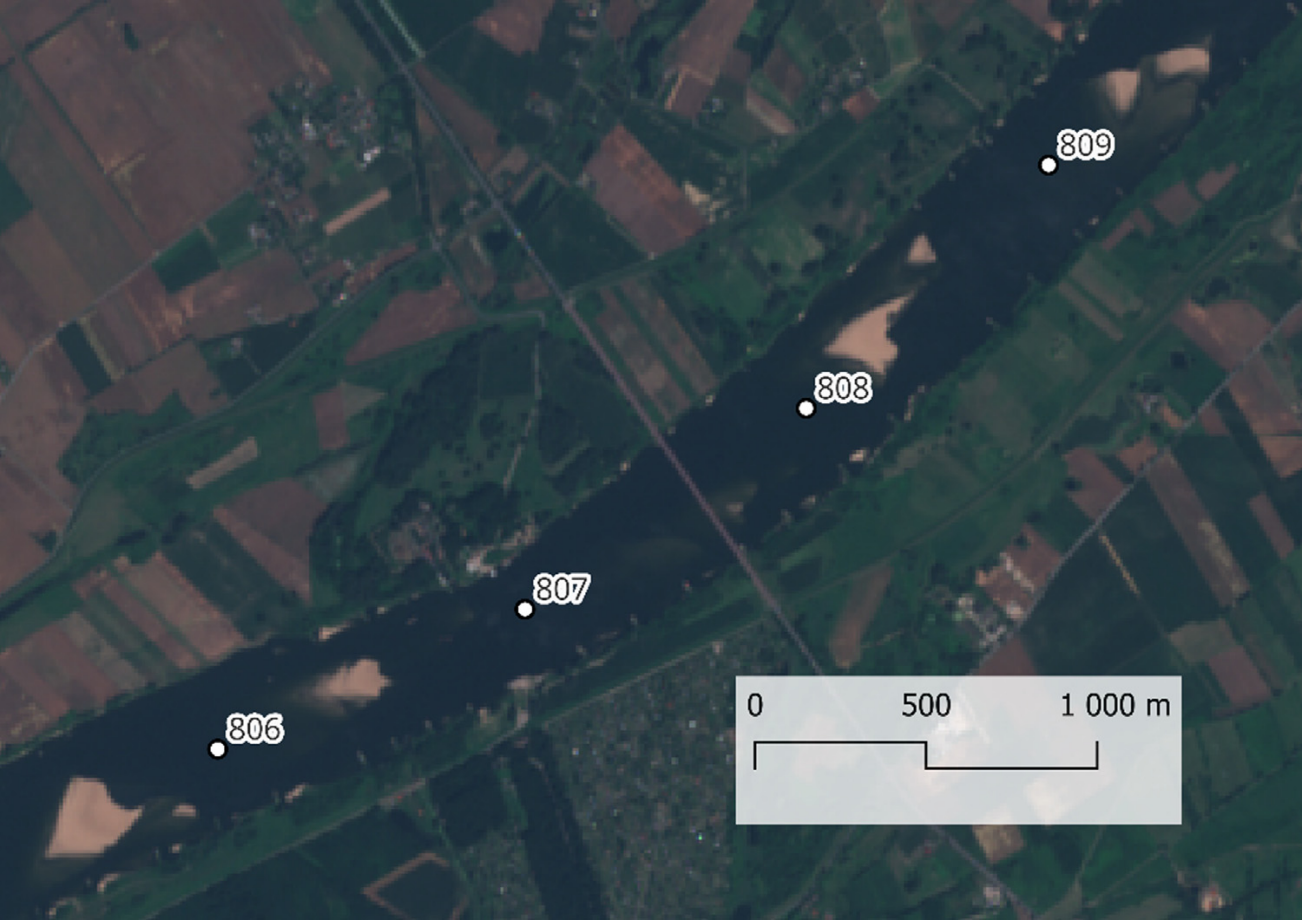
True colour, RGB combination of Satellite Sentinel-2 image.

**Fig. 7:**
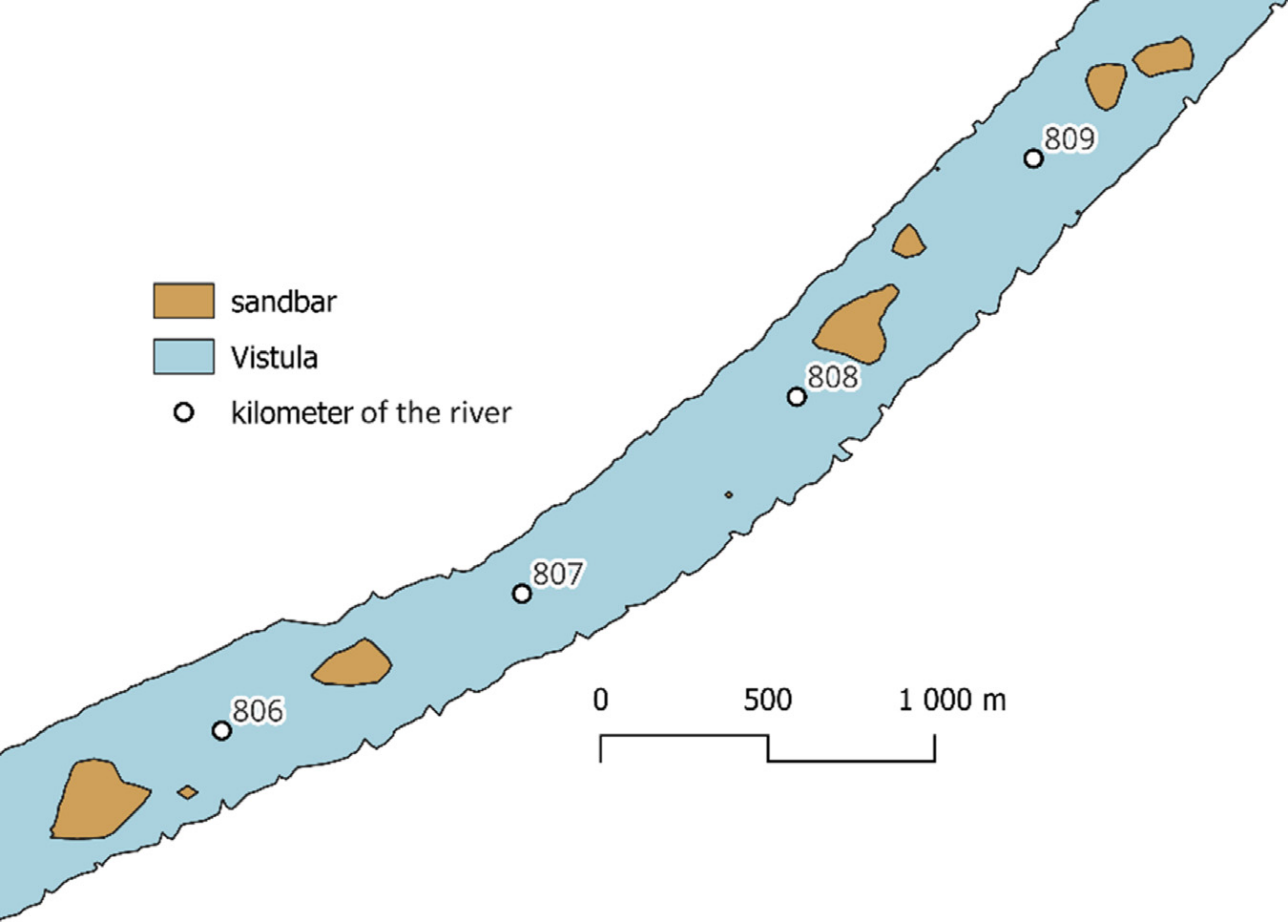
Automated generation of vectors.

The satellite image from Fig. 6 shows a section of the Vistula River between kilometres 806 and 809 in true colour composition. The image captures various riverbed forms that have emerged from the water, such as sandbars and islands. These features are visible due to the high-resolution quality of the Sentinel-2 data. The true colour composition highlights the natural colours of the river and surrounding landscape, providing a realistic view that aids in identifying and analysing riverbed forms. The subsequent figure displays the processed vector data, selected and symbolised, derived from this satellite imagery. Fig. 7

In conclusion, the Sandbar Detector plugin for QGIS is a valuable tool supporting hydrological research and water resource management. While there are various tools available for general land cover classification, such as those focused on distinguishing vegetation types or urban areas, none of these tools are specifically tailored to automate the detection of riverbed forms. Existing methods for detecting riverbed forms often rely on manual processing or general-purpose image classification tools, which are not optimized for hydrological applications and do not directly distinguish between land and water in river environments. Automating the process of detecting riverbed forms reduces the time and effort required for data analysis and increases the results' accuracy and consistency. Its simple and intuitive user interface makes the plugin accessible to a broader range of users, including those without advanced remote sensing or GIS expertise. This allows for more effective monitoring and managing of riverine environments, which is critical in dynamic hydrological changes and human activities. The plugin enables precise analysis of large datasets. It facilitates more advanced longitudinal studies, contributing to a better understanding of river processes and supporting informed decision-making regarding protecting and managing natural resources. Using the Sandbar Detector plugin, researchers and practitioners can more effectively monitor changes in river morphology, which is crucial for sustainable water management and the protection of aquatic ecosystems.

## Limitations

The only limitation is that the plugin is designed for processing Sentinel-2 Level 1C images.

## Ethics statements

This work did not involve human subjects, animal experiments, or data collected from social media platforms. Therefore, no ethical approval or informed consent was required.

## CRediT author statement

The author Klaudia Kryniecka was solely responsible for all aspects of this work, including conceptualization, methodology, software, validation, formal analysis, investigation, resources, data curation, writing – original draft, writing – review & editing, visualization, supervision, project administration, and funding acquisition.

## Declaration of competing interest

The authors declare that they have no known competing financial interests or personal relationships that could have appeared to influence the work reported in this paper.
